# Collembola of Canada

**DOI:** 10.3897/zookeys.819.23653

**Published:** 2019-01-24

**Authors:** Matthew S. Turnbull, Sophya Stebaeva

**Affiliations:** 1 Unaffiliated, Kingston, Ontario, Canada Unaffiliated Kingston Canada; 2 The Severtsov Institute of Ecology and Evolution, Russian Academy of Sciences, Leninskii pr. 33, Moscow 119071, Russia The Severtsov Institute of Ecology and Evolution Moscow Canada

**Keywords:** biodiversity assessment, Biota of Canada, Collembola, springtails

## Abstract

The state of knowledge of diversity of Collembola in Canada was assessed by examination of literature and DNA barcode data. There are 474 described extant Collembola species known from Canada, a significant change compared to the 520 species estimated to occur in Canada in 1979 (Richards 1979) and the 341 reported in the most recent national checklist (Skidmore 1993). Given the number of indeterminate or cryptic species records, the dearth of sampling in many regions, and the growing use of genetic biodiversity assessment methods such as Barcode Index Numbers, we estimate the total diversity of Collembola in Canada to be approximately 675 species. Advances in Collembola systematics and Canadian research are discussed.

Collembola, commonly known as springtails, is a class of small, entognathous, wingless hexapods that is a sister group to Insecta. They are found in most terrestrial systems and are most commonly associated with plant litter and soils, although some species are found in caves, decaying wood, tree canopies, and on the surfaces of snow and ponds. There are currently more than 8800 described Collembola species worldwide (Bellinger et al. 1996–2018). Considering that three new families and approximately 600 species have been described since 2011 (Janssens and Christiansen 2011), it is likely that many thousands of species are yet to be discovered.

The study of Collembola in Canada has a long history. Much of the work before 1979 was reviewed by Richards (1979), but additional mention should be made of the pioneering investigations of Arctic species (Wahlgren 1907, Folsom 1919, James 1938), the ground-breaking works of Hammer (1953, 1955), and further advances made during the International Biological Program (Oliver 1963, McAlpine 1964, Challet and Bohnsack 1968, Addison 1977). As noted by Richards (1979), early taxonomic and faunistic efforts created a strong foundation for modern research, but the paucity of widespread sampling in Canada makes accurate estimation of true diversity difficult. This statement remains true even today, although the situation is slowly improving.

The most important single work about Nearctic Collembola taxonomy produced since Richards (1979) is undoubtedly that of Christiansen and Bellinger (1998). The first edition of their treatment of the Collembola of North America was published in 1980–1981, the second in 1998, and continued errata and addenda have been published online up to 2003. Christiansen and Bellinger’s (1998) guide includes extensive notes, dichotomous keys, species descriptions, and distribution estimates. Despite numerous changes to species names and systematics, this work is still the most important publication for the study of Canadian collembolan taxonomy, with 840 total known species recorded for North America, and 235 species recorded for Canada (approximately 27.9% of total North American diversity).

There have been several more recent publications that have specifically focused on Canadian Collembola. The primary national list (Skidmore 1995) includes 412 species, a number often cited as total Canadian diversity; however, only 341 species in 16 families in this list were recorded in Canada, with the remainder from Alaska. While it is likely that some of the Alaskan species are also found in northwestern Canada, further sampling is needed to confirm occurrences. Additionally, several species from Skidmore’s (1995) list were later found to be synonyms, or derived from unverified records (A Babenko pers. comm.). Skidmore (1993) also published the first catalogue of type materials of collembolan species described from Canada and stored in the Canadian National Collection of Insects, Arachnids, and Nematodes (CNC). New information about type materials of 69 collembolan species at the CNC was recently published (Stebaeva et al. 2016).

Following Skidmore’s national list (Skidmore 1995), provincial and regional species lists emerged: Therrien et al. (1999) for Quebec; Cannings (2010) for British Columbia; and Lindo (2014) for prairie grasslands in Alberta. Diversity of Arctic species has been a strong area of research, with significant contributions by Fjellberg (1986) and Babenko (1994). As a result of their review, Babenko and Fjellberg (2006) were able to correct numerous synonymous and incorrect species definitions for Canadian fauna. Several new Nearctic species have been described, for example, by Rusek and Marshall (1976), Rusek (1985, 1991), Potapov (1997), Pomorski (2001), and Fjellberg (2010).

Much of the recent research on Canadian Collembola pertains to their role in agriculture, especially as indicators of soil health and as model organisms for soil toxicity assays (Behan-Pelletier 2003). Research has also been devoted to the diversity of Collembola in different forest types (e.g., Setälä et al. 1995, Puvanendran et al. 1997, Chagnon et al. 2000, Addison et al. 2003), and in response to different silviculture practices (e.g., Addison et al. 2006, Huebner et al. 2012). Some progress was made defining the paleontological history of Collembola through study of fossilized remains (Christiansen and Pike 2002). This includes ancient representatives from extinct genera of the extant families Brachystomellidae, Neanuridae s. lato, Isotomidae, Tomoceridae s. lato, and Sminthuridae s. lato, as well as the extinct family Oncobryidae (Christiansen and Pike 2002).

New collections, online resources, and genetic tools have enhanced our understanding of Collembola systematics. The leading online resource is the *Checklist of the Collembola of the World* (Bellinger et al. 1996–2018), curated by Frans Janssens. Incorporating new keys, photos, citations, species synonyms, and contact information for the expert community, it remains the most up-to-date resource for collembolan taxonomy, to which older research should be reconciled. The CNC maintains an excellent collection of about 2500 slides, including type materials of 69 species, 46 of which are from Canada (Stebaeva et al. 2016). Other collections of varying size and coverage are in academic and government institutions, including significant amounts of undetermined material.

Classification of Collembola has undergone significant changes since Richards (1979), including its elevation to class level in the subphylum Hexapoda, rather than being an order of Insecta (Bellinger et al. 1996–2018, Janssens and Christiansen 2011). Richards (1979) recognized only nine families in two orders, Arthropleona and Symphypleona; Arthropleona is now recognized as artificial and divided into the orders Entomobryomorpha and Poduromorpha, while Symphyleona was split into the orders Symphyleona and Neelipleona.

Many new families with Canadian representatives have been elevated or erected, including Tullbergiidae, Pachytullbergiidae, Odontellidae, Oncopoduridae, Tomoceridae, Orchesellidae, Seiridae, Lepidocyrtidae, Mackenziellidae, Sminthurididae, Arrhopalitidae, Katiannidae, Bourletiellidae, and Dicyrtomidae. This has been accompanied by changes at the superfamily level and corrections at lower taxonomic levels too numerous to list here. Significant systematics work continues thanks to international Apterygota colloquia (Deharveng 2004) and high level morphological study combined with genetic analyses (e.g., Schneider et al. 2011, Zhang and Deharveng 2015, Yu et al. 2016).

New genetic tools are gradually being applied to the study of collembolan phylogeny. Efforts are now being made to determine the global amount of cryptic diversity undescribed for Collembola (Cicconardi et al. 2013, Porco et al. 2014). In Canada, research using DNA barcoding to estimate species richness has been performed on Collembola from: Igloolik, Cornwallis, and Somerset islands in Nunavut (Hogg and Hebert 2004); Churchill, Manitoba (Porco et al. 2014); and northern Ontario (Telfer et al. 2015). These studies have demonstrated a high likelihood that the majority of Collembola species are undescribed; true global diversity may be an order of magnitude greater than the 50,000 global species previously estimated (Hopkin 2002, Cicconardi et al. 2013).

In addition to these peer-reviewed studies, there have been DNA barcode data submissions to the Barcode of Life Data System (BOLD) from science outreach efforts (including Bioblitz programs and the University of Guelph’s BIObus), academic researchers, and government ministries (Ratnasingham and Hebert 2007). There are a total of 70,864 specimen records from Canada in this database at the time of writing, with 1265 unique Barcode Index Numbers (BINs) representing 148 named species. Sampling has not been uniform; Ontario and British Columbia account for 48.3% and 15.8% of specimen records, respectively, whereas the territories collectively account for only 2.0% of records. Collection efforts are more likely to target larger surface-dwelling (epiedaphic) Collembola such as the family Entomobryidae (68.4% of records). Soil-dwelling (euedaphic) taxa, which are thought to be highly diverse, remain under-sampled; for example, the entire euedaphic order Poduromorpha represents only 6.1% of records. There is also a relatively high proportion of specimens that are not fully identified (41.3%) or are listed as unspecified. These data represent a tremendous opportunity for meta-analyses of collembolan distribution, diversity, species discovery rates, and the proportion of cryptic diversity.

Our research has resulted in a list of approximately 474 documented, described species (plus eight fossil species) in 23 families, compared to the 520 species in nine families estimated by Richards (1979) (Table 1), and 341 species in 16 families listed by Skidmore (1995). The 520 species reported by Richards (1979) represented an estimate of the total fauna, known and unknown. The actual number of described species known from Canada in 1979 is unknown as Richards did not publish a checklist. However, in the first edition of *The Collembola of North America North of the Rio Grande*, Christiansen and Bellinger (1980–1981) reported 195 described species from Canada. Thus, over the last nearly 40 years, the described fauna of Canada has increased by approximately 143%.

**Table 1.** Census of Collembola in Canada. Information sources for all families are Bellinger et al. (1996–2018), Christiansen and Bellinger (1998), and Deharveng (2004).

Taxon^1^	No. species reported by Richards (1979)	No. species currently reported from Canada	No. BINs available for Canadian species^2^	Est. no. undescribed or unrecorded species in Canada	General distribution by ecozone^3^
**Order Poduromorpha**					
**Superfamily Poduroidea**					
Poduridae	1	1	5	1–3	most ecozones
**Superfamily Hypogastruroidea**					
Hypogastruridae	65	71	98	18–22	most ecozones
**Superfamily Onychiuroidea**					
Onychiuridae ^4^	50	36	36	16–17	most ecozones
Tullbergiidae	?^4^	20	6	6	most ecozones
Pachytullbergiidae	?^4^	1	0	0	Pacific Maritime
**Superfamily Neanuroidea**					
Brachystomellidae ^5^	22	2	6	0	Montane Cordillera, Pacific Maritime, Western Interior Basin
Neanuridae ^6^	65	57	91	35–39	most ecozones
Odontellidae	?^5^	9	22	4	most ecozones
**Order Entomobryomorpha**					
**Superfamily Isotomoidea**					
Isotomidae	120	141	258	40–62	most ecozones
**Superfamily Tomoceroidea**					
Oncopoduridae	?^7^	1	0	0	Pacific Maritime
Tomoceridae	?^7^	12	70	0	most ecozones
**Superfamily Entomobryoidea**					
Orchesellidae	?^7^	11	0	1	most ecozones
Seiridae	?^7^	1	0	0	Pacific Maritime
Lepidocyrtidae	?^7^	19	0	3–4	most ecozones
Entomobryidae ^7^	80	32	240	11–16	most ecozones
**Order Neelipleona**					
Neelidae	2	3	17	3	most ecozones
**Order Symphypleona**					
**Superfamily Sminthuridoidea**					
Mackenziellidae	? ^8^	1	0	0	southern Arctic, Taiga Plains
Sminthurididae	?^8^	12	78	13	most ecozones
**Superfamily Katiannoidea**					
Arrhopalitidae	?^8^	11	16	7	most ecozones, few records in Arctic
Katiannidae	?^8^	10	87	4	most ecozones
**Superfamily Dicyrtomoidea**					
Dicyrtomidae	?^8^	8	78	3	most ecozones, few records in Arctic and Taiga ecozones
**Superfamily Sminthuroidea**					
Sminthuridae ^8^	115	8	23	5–6	most ecozones, few records in Arctic and Taiga ecozones
Bourletiellidae	?^8^	7	133	8	most ecozones
**Total**	**520**	**474**	**1265**	**180–204**	

^1^Classification follows (Bellinger et al. 1996–2018). ^2^All data are from BOLD (Ratnasingham and Hebert 2007) and current as of April 4, 2018. Data are Barcode Index Numbers (BINs), as defined in Ratnasingham and Hebert (2013). ^3^See figure 1 in Langor (2019) for a map of ecozones. ^4^The definition of Onychiuridae used by Richards (1979) likely included members of the modern Tullbergiidae. ^5^Richards (1979) very likely followed Salmon (1964) and included in Brachystomellidae some species of the modern family Odontellidae. ^6^Richards (1979) reported 65 species of Pseudachorutidae, which is now a subfamily of Neanuridae. ^7^Richards (1979) undoubtedly included in Entomobryidae some species of the modern families, Oncopoduridae, Tomoceridae, Orchesellidae, Seiridae, and Lepidocyrtidae. ^8^Richards’ concept of Sminthuridae undoubtedly included species currently placed in Sminthurididae, Arrhopalitidae, Katiannidae, Dicyrtomidae, and Bourletiellidae.

Distributions of Collembola species in Canada are difficult to determine as many specimens come from only a single location and species may be entered onto provincial lists with few and/or questionable records. There is also a high likelihood that seemingly widely distributed species only appear cosmopolitan due to morphological convergences with disparate species, and such problems will be best solved by study of morphological and genetic characters. Despite the challenges with delimiting species ranges, the majority of Canadian Collembola families are thought to be widely distributed, with representatives in most ecozones. There are, however, cases of region-specific records at lower taxonomic levels. From available published records, there are three genera currently recorded only in the Atlantic Maritime ecozone and several other species are known from only one jurisdiction: Manitoba (7 spp.), Alberta (11), Nunavut (15), Quebec (30), and Ontario (30) (e.g., Skidmore 1995, Christiansen and Bellinger 1998, Therrien et al. 1999, Babenko and Fjellberg 2006, Lindo 2014). British Columbia contains the most species not recorded in other areas; three families, 22 genera, and 103 of the 248 species recorded in the province have not been recorded from other Canadian jurisdictions (Cannings 2010). Species found only in one ecozone or jurisdiction should not be construed as endemic but may only appear as such simply because there is insufficient knowledge about distribution. For example, there are only 13 species records published from New Brunswick, five from Saskatchewan, and none from Prince Edward Island (e.g., Skidmore 1995, Christiansen and Bellinger 1998). This apparent lack of diversity is more reflective of a paucity of available regional expertise and sampling effort than true distributions.

We estimate there are approximately 180–204 existing records of Collembola in Canada, which were either not described to species level or were misidentified as existing species when they may in fact represent undocumented species. We consider this to be a conservative estimate of the undocumented Canadian springtail fauna. However, it is likely that there is a high number of cryptic species that will require advanced genetic techniques to differentiate (e.g., Cicconardi et al. 2013, Porco et al. 2014). For example, given there are 1265 BINs associated with the 148 specimens assigned species names on BOLD, and that theorized interspecies divergences for Collembola range from 8% (Hogg and Hebert 2004) to 14% (Porco et al. 2014), many of these named specimens are likely to represent multiple morphologically cryptic species. There is a ratio of approximately 8.5 BINs per identified springtail species in BOLD. If a similar ratio of BINs per described species is assumed for the 474 documented Collembola species in Canada, there would be approximately 4051 BINs expected for the currently described national fauna. If it is conservatively assumed that 80% of these BINs represent distinct species, it is possible that there are up to 3240 Collembola species in the Canadian fauna, meaning that over 2700 species have yet to be described. Although this estimate is based on several assumptions, we consider it to be reasonable given that a global fauna of 65,000 species of Collembola was estimated based on BIN data (Porco et al. 2014). We expect much of this undiscovered diversity to lie in under-sampled euedaphic taxa in the Maritimes and northern interiors of the western provinces. Collembola is a group full of opportunities for aspiring researchers, and there is serious need of a new generation of taxonomists who can integrate both morphological and genetic data.
